# Validation of an educational manual for breast cancer patients undergoing radiotherapy*

**DOI:** 10.1590/1518-8345.3197.3384

**Published:** 2020-10-19

**Authors:** Flávia Oliveira de Almeida Marques da Cruz, Edison Tostes Faria, Paula Elaine Diniz dos Reis

**Affiliations:** 1 Universidade de Brasília, Brasília, DF, Brazil.; 2 Centro Universitário do Distrito Federal, Brasília, DF, Brazil.; 3 Universidade de Brasília, Faculdade de Medicina, Brasília, DF, Brazil.; 4 Universidade de Brasília, Faculdade de Ciências da Saúde, Brasília, DF, Brazil.

**Keywords:** Oncology Nursing, Health Education, Nursing Care, Validation Study, Educational Technology, Breast Neoplasms, Enfermagem Oncológica, Educação em Saúde, Cuidados de Enfermagem, Estudo de Validação, Tecnologia Educacional, Neoplasias da Mama, Enfermería Oncológica, Educación en Salud, Atención de Enfermería, Estudio de Validación, Tecnología Educacional, Neoplasias de la Mama

## Abstract

**Objective:**

to validate the content and appearance of an educational manual for breast cancer patients undergoing radiation therapy.

**Method:**

methodological research, which had the Theory of Psychometry as a theoretical-methodological reference. The minimum 80% Concordance Index was considered to ensure the adequacy of the material. The sample consisted of 17 experts in the subject area of the educational manual and 12 patients previously submitted to radiotherapy due to the diagnosis of breast cancer.

**Results:**

two items of the expert evaluation tool were found to have a concordance index <80%. The other items were considered adequate and/or totally adequate in the three blocks of analysis proposed for the experts: objectives - 89.07%, structure and presentation - 92.94%, and relevance - 93.13%; and good and/or very good in the five blocks of analysis proposed for the patients: objectives, organization, writing style, appearance, and motivation, all with 100% agreement rate.

**Conclusion:**

the educational manual, after having been perfected based on the suggestions of the sample and the scientific literature, was considered valid according to its content and appearance, suggesting its contribution to the clinical practice of nursing and to the understanding of the treatment to which patients with breast cancer are submitted.

## Abstract (pt)

**Objetivo:**

validar o conteúdo e a aparência de um manual educativo para pacientes com câncer de mama submetidas à radioterapia.

**Método:**

pesquisa metodológica, que teve a Teoria da Psicometria como referencial teórico-metodológico. Foi considerado o Índice de Concordância mínimo de 80% para garantir a adequação do material. Compuseram a amostra 17 peritos na área temática do manual educativo e 12 pacientes previamente submetidas à radioterapia devido ao diagnóstico de câncer de mama.

**Resultados:**

dois itens do instrumento de avaliação destinados aos peritos obtiveram *Índice de Concordância* <80%. Os demais itens foram considerados adequados e/ou totalmente adequados nos três blocos de análise propostos para os peritos: objetivos - 89,07%, estrutura e apresentação - 92,94%, e relevância - 93,13%; e bom e/ou muito bom nos cinco blocos de análise propostos para as pacientes: objetivos, organização, estilo da escrita, aparência, e motivação, todos com Índice de Concordância de 100%.

**Conclusão:**

o manual educativo, após ter sido aperfeiçoado com base nas sugestões da amostra e na literatura científica, foi considerado válido segundo o conteúdo e aparência, sugerindo sua contribuição para a prática clínica da enfermagem e para a compreensão do tratamento, ao qual pacientes com câncer de mama são submetidas.

## Abstract (es)

**Objetivo:**

validar el contenido y la apariencia de un manual educativo para pacientes con c*áncer* de mama sometidas a radioterapia.

**Método:**

investigación metodológica, que utilizó la Teoría de la Psicometría como referencial teórico metodológico. Fue considerado el *Índice de C*oncordancia *mínimo de ٨0% para garanti*zar la adecuación del material. Compusieron la muestra 17 peritos en el *área temática* del manual educativo y 12 pacientes previamente sometidas a radioterapia debido al diagnóstico de c*áncer* de mama.

**Resultados:**

dos *ítems* del instrumento de evaluación destinado a los peritos obtuvieron *Índice de C*oncordancia <80%. Los demás *ítems* fueron considerados adecuados y/o totalmente adecuados en los tres bloques de análisis propuestos para los peritos, obteniéndose los siguientes resultados: objetivos 89,07%; estructura y presentación 92,94%; y, relevancia 93,13%. En los cinco bloques de análisis propuestos para las pacientes se obtuvo bueno y/o muy bueno, con *Índice de* Concordancia de 100% para: objetivos, organización, estilo de escritura, apariencia, y motivación.

**Conclusión:**

el manual educativo, después de haber sido perfeccionado con base en las sugerencias de la muestra y en la literatura científica, fue considerado válido según contenido y apariencia, sugiriendo que puede contribuir para la práctica clínica de la enfermería y para la comprensión del tratamiento al cual son sometidas las pacientes con c*áncer* de mama.

## Introduction

Regardless of cases of non-melanoma skin cancer, breast cancer is the most common type of cancer in women, with approximately 2.1 million new cases diagnosed in 2018. The incidence has increased in most regions of the world and in Brazil there were 16,724 deaths in 2017 due to this disease, which could reach more than 66,000 new women for each year of the triennium 2020-2022^(^ 1 ^)^.

When the breast region is submitted to radiotherapy, the most common effects are pain, skin alterations, also known as radiodermatitis, restriction of mobility, local sensory alteration and fatigue^(^ 2 ^)^. It is essential to guide breast cancer patients undergoing radiotherapy about the possible adverse effects related to this therapy, and knowledge about the ways to prevent or minimize such effects is essential.

Besides the physical and systemic factors related to the disease and treatment, it is worth mentioning the important psychological impact caused by the disease, mainly on the aesthetics of the body image, sexuality and femininity of women with breast cancer^(^ 3 ^)^. Therefore, it is essential that the nurse performs his or her assistance role related to the disease and therapy, as well as acting as a facilitator in the coping process by providing integral and individualized care^(^ 4 ^)^.

Therefore, it is necessary to develop health technologies aimed at breast cancer patients undergoing radiotherapy, with the objective of transmitting knowledge and teaching regarding the guidelines and behaviors necessary for the management of the adverse effects of therapy.

Informational manuals are important strategies to support educational activities, as they facilitate the work of the health team by standardizing and improving patient understanding^(^ 5 ^)^. In view of this reality, the conduct of the nurse can be systematized through the use of a guidance manual, which should be effective in promoting information regarding treatment and self-care at home, as well as being important for controlling the adverse effects of radiotherapy^(^ 4 ^)^. The printed material can help the patient memorize information, which favors the work of the nurse in health education activities^(^ 6 ^)^.

Submitting an instrument or tool to the validation process before its use is essential in order to verify the real quality of the data and information transmitted^(^ 7 ^)^. Therefore, before making a professional tool available for use, it is essential to evaluate it in order to know its effectiveness^(^ 8 ^-^ 9 ^)^.

Taking into consideration the importance of ensuring the validity of the material before its use, the objective of this study was to validate the content and appearance of an educational manual intended for breast cancer patients undergoing radiotherapy.

## Method

This is a methodological research, focusing on the development, evaluation and improvement of a methodological strategy: an educational manual for breast cancer patients submitted to radiotherapy. The preparation of the material began with a review of the literature on breast cancer, radiotherapy, its adverse effects and the care needed to prevent them, in addition to other relevant topics on the subject. The guidelines of an educational manual for head and neck cancer patients undergoing radiotherapy were considered as a model^(^ 4 ^,^ 10 ^)^, with the appropriate modifications and adaptations related to the subject matter addressed in this study.

The manual was called “Orientations Manual: Breast Radiotherapy”, and was addressed to the patients seen at the Radiotherapy Outpatient Clinic of the Unit of High Complexity in Oncology of the *Hospital Universitário de Brasília* (UNACON/HUB), Brasília, DF, Brazil, which offers multidisciplinary outpatient care to people diagnosed with cancer. The material is 148x210 mm in size and contains 36 pages divided into pretextual items (cover, back cover, cataloguing sheet, index, presentation and registration card), textual items (chapters on radiotherapy, stages of treatment, adverse effects of radiotherapy and how to prevent them) and post-textual items (latest information, weekly diary and bibliographical references).

In general, the material includes information related to breast radiotherapy, such as equipment used and steps to be taken, and the adverse effects involved, such as radiodermatitis. In addition to this information, there are also guidelines related to skin care, such as the use of products, creams, perfume and deodorant in the region exposed to radiation, as well as their sanitation, and care related to hydration and food. Finally, the educational manual has a weekly diary, in which users can record the signs and symptoms perceived throughout the week, as well as the care given.

The content validation indicates to what extent a tool is adequate in relation to the approach of the information and knowledge involved, with the aim of testing the content and checking its adequacy according to the subject of interest. Therefore, at least six experts in the subject area of the material must be able to evaluate it individually, granting it validity. The appearance validation, on the other hand, aims to verify that the manual is understandable to the target population, if it is clear, easy to read and understand, being based on the judgment of those who will use the instrument^(^ 11 ^-^ 13 ^)^.

The professionals involved were selected from contacts in the research group of which the researchers of this study are part, through analysis of the Curriculum Lattes of people who perform activities in the thematic field addressed in the educational manual. Intentional non-probabilistic sampling was used, since it excludes randomness in sample selection, which must be defined by means of requirements deemed necessary for its participation^(^ 14 ^)^. The factors used to choose these participants were then determined according to their degree, specialization, scientific production, knowledge and time of work on the topic involved^(^ 15 ^)^.

The invitation was sent by email to 28 professionals qualified to participate in the survey. One of the professionals expressed unwillingness to participate and ten did not respond to the invitation, even after three attempts to contact them and, therefore, were not included in the study. Once accepted, the materials corresponding to the evaluation were sent, also by e-mail, which were the Free and Informed Consent Term, the evaluation instrument and the educational manual to be evaluated.

The data was collected using a Likert scale evaluation instrument, with five levels of judgment regarding the items: inadequate (I), partially adequate (PA), not sure (N), adequate (A) and totally adequate (TA).

The validation of the educational manual was also based on the opinion of women over 18 years of age, with the ability to understand the material, willingness to participate in the evaluation process and who had completed the radiotherapy treatment due to the diagnosis of breast cancer no more than two months prior. The latter criterion was necessary to ensure that the participant had sufficient knowledge and experience to assess the aspects addressed in the material.

The patients selected from the UNACON/HUB database were invited by phone call and, after acceptance, attended the institution at a previously agreed date and time. 20 women were contacted, of whom three were unavailable for participation and five did not show up at the agreed date and time, even after three scheduling attempts and were thus not included in the study. During the meeting, the research guidelines were again offered and, upon confirmation of acceptance and signature of the Term of Free and Informed Consent, the participants received the educational manual and the evaluation tool.

The data was collected by means of an evaluation instrument, also prepared on a Likert scale, with five levels of judgment regarding the items: bad (B), regular (R), not sure (N), good (G), and very good (VG).

The data collection took place from October to December 2017. To attest the validity of each item addressed in the evaluation instruments, the minimum Concordance Index (CI) of 80% was determined among participants. The set composed of options A and TA should obtain at least 80% of expert responses, and the group of options G and VG should also obtain at least 80% of patient responses, to ensure that the educational manual is considered valid. The items that obtained CI below 80% were analyzed and the educational manual was improved according to suggestions of the participants, scientific literature and clinical evidence. The information was tabulated and processed by means of descriptive analysis.

The research project was sent to the Research Ethics Committee of the College of Health Sciences of the University of Brasilia (CEP/FS-UnB), approved by Opinion No. 493.456, CAAE: 24592213.1.0000.0030.

## Results

For content validation, the sample consisted of 17 professionals: nine nurses, four physicians, two physicists and two psychologists. Regarding the degree, one had the title of post-doctorate, seven of doctor, 16 of master and 14 of specialist, emphasizing that a single person could have more than one title. Regarding sex, 11 were women and six were men. Age ranged from 27 to 63 years (mean (M): 38 years; standard deviation (SD): 9), while time of training ranged from five to 39 years (M: 14; SD: 9) and that of working in the subject area of the educational manual ranged from three to 30 years (M: 12; SD: 7). Regarding the current occupation, 12 were engaged in welfare activities and five were teachers and researchers.

For appearance validation, the sample consisted of 12 patients previously submitted to radiotherapy due to the diagnosis of breast cancer. According to schooling, two participants studied until elementary school, seven studied until high school and three participants attended higher education. Age ranged from 32 to 53 years (M: 47 years; PD: 6). Regarding skin colour, seven declared themselves as brown, three as black and two as white. About marital status, ten were married, one divorced and one was single. Only two participants were from Goiás, while the others were from the Federal District.

The evaluation instrument for the experts was composed of three blocks of analysis: objectives; structure and presentation; and relevance; while the instrument for patients previously submitted to radiotherapy due to the diagnosis of breast cancer was composed of five blocks of analysis: objectives; organization; writing style; appearance; and motivation. Regarding the validation process of content and appearance of the educational manual, the opinion of the experts and patients was analyzed in a quantitative way, through the answers given to the items in the evaluation instruments.

Table 1 shows the expert responses and the Concordance Index (CI) for each item of the first evaluation block. Items E and G did not reach the established minimum CI, both with 70.58% agreement among experts. All other items in the block reached the CI of 80%, ranging from 88.23 to 100%, with the average CI of the block being 89.07%.

**Table 1:** Expert evaluation of the objectives of the education manual. Brasília, DF, Brazil, 2017

Evaluation items		n=17				%^¶^
	I*	PA^†^	N^‡^	A^§^	TA^||^	
A. The material is consistent with the needs of breast cancer patients undergoing radiotherapy	0	0	0	6	11	100.00
B. It is consistent from the point of view of the treatment process (stages of radiotherapy)	0	0	0	4	13	100.00
C. It is coherent from the point of view of the health education process (it provides important and necessary information and guidance)	0	0	0	6	11	100.00
D. It is effective for the maintenance of self-care at home by the patient	0	0	2	6	9	88.23
E. Can promote changes in attitude and behavior	0	0	5	3	9	70.58
F. It can circulate in the scientific environment of oncology and radiotherapy	0	0	1	5	11	94.11
G. Meets the objectives of UNACON/HUB** and other institutions that work with cancer and radiotherapy, and its use can be extended to other health services	0	3	2	4	8	70.58
**Total**	0	3	10	34	72	89.07

* I = Inadequate;

† PA = Partially adequate;

‡ N = Not sure;

§ A = Adequate;

|| TA = Totally adequate;

¶ % = Concordance Index calculated by the sum of adequate and totally adequate judgments: TA+A x 100 / total of responses;

** UNACON/ HUB = High Complexity Unit in Oncology of the University Hospital of Brasilia

Table 2 presents the answers given by the experts, as well as the CI of each item in the second evaluation block, in which the items reached the minimum CI of 80%, ranging from 82.35 to 100%, with the average CI of the block being 92.94%.

**Table 2:** Expert evaluation of the structure and presentation of the education manual. Brasília, DF, Brazil, 2017

Evaluation items		n=17				%^¶^
	I*	PA^†^	N^‡^	A^§^	TA^||^	
A. The material is suitable for women with breast cancer who have undergone radiotherapy (target public)	0	0	0	6	11	100.00
B. The material is able to reach different socio-cultural layers	0	1	2	5	9	82.35
C. The information is presented in a clear and objective manner	0	0	0	4	13	100.00
D. The information presented is scientifically correct	0	0	0	3	14	100.00
E. There is a logical sequence in the content covered	0	1	1	2	13	88.23
F. The information is well structured in concordance and spelling	0	0	0	5	12	100.00
G. The writing style is able to reach different socio-cultural layers	0	1	2	7	7	82.35
H. The size of the title and topics is adequate	0	0	0	4	13	100.00
I. The illustrations are adequate and in sufficient amount	0	1	1	5	10	88.23
J. The amount of information and guidance is adequate	0	1	1	7	8	88.23
**Total**	0	5	7	48	110	92.94

* I = Inadequate;

† PA = Partially adequate;

‡ N = Not sure;

§ A = Adequate;

|| TA = Totally adequate;

¶ % = Concordance Index calculated by the sum of Adequate and Totally Adequate judgments: TA+A x 100 / total answers

Table 3, in turn, presents the responses given by the experts and the CI for each item of the third evaluation block. All items reached the minimum CI of 80%, ranging from 86.66 to 100%, with the average CI of the block being 93.13%.

**Table 3:** Expert evaluation of the relevance of the education manual. Brasília, DF, Brasil, 2017

Evaluation items		n=17				%^¶^
	I*	PA^†^	N^‡^	A^§^	TA^||^	
A. The topics covered portray aspects that are essential to self-care and which should be reinforced for the target audience	0	0	0	5	12	100.00
B. The manual allows the transfer and generalizations of learning in different contexts (hospital and home)	0	0	0	6	11	100.00
C. The manual is effective when it proposes to the patient to acquire knowledge to perform self-care at home	0	0	3	3	11	86.66
D. The manual is effective when it proposes to the patient the acquisition of information about the treatment process (steps of radiotherapy)	0	0	3	3	11	86.66
E. Addresses the most pertinent issues for the breast cancer patient in radiotherapy	0	0	0	9	8	100.00
F. It is suitable for use as a form of educational technology in the practice of health professionals	0	1	0	6	10	93.33
**Total**	0	1	6	32	63	93.13

* I = Inadequate;

† PA = Partially adequate;

‡ N = Not sure;

§ A = Adequate;

|| TA = Totally adequate;

¶ % = Concordance Index calculated by the sum of Adequate and Totally Adequate judgments: TA+A x 100 / total answers

Table 4 presents the answers given by the patients and the CI of each item of the five blocks of analysis of the evaluation instrument intended for this sample. All items achieved 100% CI, with the average CI of the block also being 100%.

**Table 4:** Evaluation of patients previously submitted to radiotherapy due to the diagnosis of breast cancer. Brasília, DF, Brazil, 2017

Evaluation items	n=12					%^¶^
	B*	R^†^	N^‡^	G^§^	VG^||^	
Objectives						
A. The material meets the goals of women with breast cancer who undergo radiotherapy	0	0	0	2	10	100.00
B. The material brings the main needs of women with breast cancer during radiotherapy	0	0	0	2	10	100.00
C. Can help during treatment	0	0	0	2	10	100.00
D. Can help with home care	0	0	0	2	10	100.00
**Total**	0	0	0	8	40	100.00
Organization						
A. The initial section indicates the content of the material	0	0	0	2	10	100.00
B. The size of the title and content is adequate	0	0	0	2	10	100.00
C. The topics have a logical sequence for learning	0	0	0	2	10	100.00
D. The amount of information is adequate	0	0	0	2	10	100.00
E. The topics covered portray key aspects	0	0	0	2	10	100.00
F. The material is appropriate and arouses the woman's interest in its use	0	0	0	2	10	100.00
**Total**	0	0	0	12	60	100.00
Writing style						
A. The writing is in proper style	0	0	0	2	10	100.00
B. The text is interesting and the tone is friendly	0	0	0	2	10	100.00
C. Vocabulary is accessible and easy to understand	0	0	0	2	10	100.00
D. The theme of each session is linked to its text	0	0	0	2	10	100.00
E. The text is clear	0	0	0	2	10	100.00
F. The text is in sufficient quantity	0	0	0	2	10	100.00
**Total**	0	0	0	12	60	100.00
Appearance						
A. The sections of the material have a logical sequence	0	0	0	2	10	100.00
B. The illustrations are simple and informative	0	0	0	2	10	100.00
C. The illustration is important to complement the text	0	0	0	2	10	100.00
D. The illustrations are realistic enough	0	0	0	2	10	100.00
E. The illustrations are in sufficient quantity	0	0	0	2	10	100.00
**Total**	0	0	0	10	50	100.00
Motivation						
A. The material is suitable for the age, gender and culture of the target population	0	0	0	2	10	100.00
B. The material is logical	0	0	0	2	10	100.00
C. The interaction between the woman and the material is possible, encouraging actions of care	0	0	0	2	10	100.00
D. The material addresses the issues needed for women with breast cancer who undergo radiotherapy	0	0	0	2	10	100.00
E. Can help promote a change in attitude	0	0	0	2	10	100.00
F. The material offers women the opportunity to acquire the knowledge to perform self-care	0	0	0	2	10	100.00
**Total**	0	0	0	12	60	100.00

* B = Bad;

† R = Regular;

‡ N = Not sure;

§ G = Good;

|| VG = Very good;

¶ % = Concordance Index calculated by the sum of good and very good judgments: MB+B x 100 / total

## Discussion

In relation to the first block of the evaluation instrument for experts (Table 1), which sought to identify the opinion of professionals regarding the objective of the educational manual, it was possible to verify that it was considered valid in terms of its capacity to achieve the purposes and goals for which it was proposed, since the block as a whole achieved an 89.07% Concordance Index. However, items E and G need to be taken into consideration, since they have not individually reached the minimum CI required.

Item E, which analyses the educational manual in relation to its capacity to promote changes in behavior and attitude, reached CI of 70.58%, considering that five experts chose the option “not sure” for such item. Experts are unsure about the material’s ability to generate behavior and attitude change in isolation, given the important role of the nurse during nursing appointments.

Considering the proximity between nurse and patient, this professional is able to meet the physiological and clinical demands, as well as those related to psychological and social issues, with positive results in the association of interventions performed by nurses and the achievement of benefits in physical and emotional well-being, reducing anxiety and stress and improving the insomnia of patients^(^ 16 ^)^.

The objective of this research is, therefore, to provide a validated educational manual as a support strategy to be used as a guide to subsidize nursing care provided to patients with breast cancer undergoing radiotherapy. In this way, the manual is part of an educational follow-up work done by the nurse, therefore, it should be a tool used concomitantly with the work process of this professional, and not in an isolated manner.

The different professionals who participated as experts in this study perform activities in the radiotherapy outpatient clinic of UNACON/HUB or in other institutions distributed throughout Brazil, such as Rio de Janeiro, São Paulo, Minas Gerais, Paraíba and Ceará. This distribution was important to evaluate the suitability of the educational manual for other services that also work with radiotherapy, and therefore to verify if its use could be extended to institutions beyond UNACON/HUB. In addition, the participation of different professional categories is very favorable to the validation process, since it makes it possible to bring together different opinions specialized in the subject matter addressed by the material, taking into account the specificities of each profession^(^ 17 ^)^.

The item G, which analyses whether the educational manual meets the objectives of UNACON/HUB and other institutions working with radiotherapy, reached a CI of 70.58%, considering that two experts chose the option “not sure” and three chose the option “partially suitable” for that specific item. These experts have clarified their choice due to the particularities present in the services of different regions of the country. Even though it has been judged to be accessible to various socio-cultural layers, there are specificities arising from routine, planning techniques and care related to radiotherapy in each health institution, which may make the applicability of the educational manual in other services unfeasible, as observed in a previous study^(^ 4 ^)^. Therefore, the material should be adapted according to each service so that it can be used in other institutions that differ in treatment and/or population from UNACON/HUB.

It is essential to adopt clear language that is accessible to all layers of society, since the educational manual needs to be easy to understand. The information selected to be included in the material must be that which is really indispensable for it to be significant, attractive, concise and objective. It is also important to use images and photos correlated with textual information, as a way of illustrating the educational manual, stimulating its reading and facilitating its understanding, since they transform textual information into visual language^(^ 18 ^-^ 19 ^)^.

Therefore, the educational manual needs to be planned and developed for the needs of the population, as a way to foster interest and understanding on the part of those who will benefit from the material. The language used must be clear, succinct and appropriate to the educational and cultural level of the population, and may be supported by illustrations and images, using the playful medium to promote communication, arouse interest and motivate the use of material^(^ 20 ^)^.

In relation to the changes made, the most frequent considerations pointed out by the experts were related to changing vocabulary in order to make the reading of the educational manual easier, more objective and clear, thus simplifying the understanding by the target public.

Important information that was included in the material concerns the non-use of deodorant by patients undergoing radiotherapy treatment in the breast region. This suggestion was taken up, as metal-based compounds such as magnesium, aluminum or zinc are generally present in antiperspirants. There is a concern about the increase in the superficial dose on the skin caused by a bolus effect of the deodorant, due to its aluminum composition^(^ 21 ^-^ 22 ^)^.

In the scientific literature, there is evidence suggesting the use of deodorant by women who receive radiation for breast cancer, regardless of whether the product is composed of aluminum or not, in view of the concern of women who do not use deodorant in relation to the appearance of possible unpleasant odors^(^ 21 ^-^ 22 ^)^.

However, there is no consensus and further studies are needed on the safety of using antiperspirants containing aluminum in their composition. Therefore, considering also the institutional routine already established at UNACON/HUB to avoid the use of this product during radiotherapy, such conduct was maintained in the educational manual.

The suggestion to include information on lymphedema has not been accepted, since such an effect is related to a chronic condition experienced after treatment, mainly due to axillary lymph nodes dissection associated with regional lymph node irradiation, and does not concern the adverse effects commonly encountered during radiotherapy^(^ 23 ^)^. The suggestion to add information on late treatment reactions, such as fibrosis and adherence, was also not accepted, since the proposed material is intended to accompany the patient during radiotherapy and guide her through the acute reactions of treatment.

The inclusion of too much information that is not essential for the moment experienced by patients can generate unnecessary anxiety and fear. Aspects related to the strategy of presenting the guidelines of a teaching material are very important, since the message conveyed must have credibility and confidence, and be appropriate to the context of the target audience, using only the information considered essential for a correct understanding of the text^(^ 10 ^)^. Therefore, the ideal is to highlight the indispensable and useful information for the self-care of the patient during the treatment.

Thus, the final result of the elaboration and improvement of the educational manual was a material with essential information for the patient with breast cancer in radiotherapy, besides also containing illustrations coherent with the text, favoring the communication and the understanding of those who use the material.

From the moment the patient takes home a material containing the guidelines that were provided during the nursing consultation, it is possible to expand such information outside the hospital environment, disseminating it at home between the caregivers and the relatives that relate to the patient, since the patient has the material for constant consultation, in case of doubts or wishes^(^ 4 ^)^.

Therefore, the experts’ responses were broadly in agreement. All three analysis blocks obtained a concordance index (CI) above the 80% established. Regarding the responses of patients previously submitted to radiotherapy due to the diagnosis of breast cancer, the following were also concordant. All five blocks of analysis obtained a 100% Concordance Index (CI), exceeding the expectations of acceptability of the material by women who have already undergone radiotherapy. In addition, one can see in the reports of such participants the great satisfaction with the material evaluated.

It is important to note that the content of the educational manual must be constantly updated based on scientific innovations and changes that may arise in the demands presented by breast cancer patients undergoing radiotherapy.

The limitation of this study is related to the fact that the apparent validation was performed by 12 women with breast cancer previously undergone radiotherapy, an apparently small number. However, this limitation can be justified by the fact that women from different levels of education were included, with participants who studied up to primary school, others who studied up to high school, and also participants who studied in higher education.

## Conclusion

The educational manual was prepared from a literature review and adaptations made in an educational manual validated in a previous study, with the appropriate modifications and adaptations related to the theme, and was improved based on the suggestions of the research participants and scientific literature. It was validated according to content and appearance, whose evaluation process included 17 experts in the thematic area of the material and 12 patients previously submitted to radiotherapy due to the diagnosis of breast cancer.

For the expert evaluation tool, two items obtained a Concordance Index <80% The other items were considered adequate and/or totally adequate in the three blocks of analysis proposed for the experts: objectives - 89.07%, structure and presentation - 92.94%, and relevance - 93.13%; and good and/or very good in the five blocks of analysis proposed for the patients: objectives, organization, writing style, appearance, and motivation, all with a 100% Concordance Index.

It is suggested that the educational manual may contribute to the clinical practice of nursing and to the understanding of the therapy to which breast cancer patients are subjected when undergoing radiotherapy.
